# Metabolomic Variability of Different Soybean Genotypes: β-Carotene-Enhanced (*Glycine max*), Wild (*Glycine soja*), and Hybrid (*Glycine max* × *Glycine soja*) Soybeans

**DOI:** 10.3390/foods10102421

**Published:** 2021-10-13

**Authors:** Jung-Won Jung, Soo-Yun Park, Sung-Dug Oh, Yejin Jang, Sang-Jae Suh, Soon-Ki Park, Sun-Hwa Ha, Sang-Un Park, Jae-Kwang Kim

**Affiliations:** 1 Division of Life Sciences, Incheon National University, Incheon 22012, Korea; 19961015@inu.ac.kr; 2 National Institute of Agricultural Sciences, Rural Development Administration (RDA), Jeonju-si 55365, Korea; psy22@korea.kr (S.-Y.P.); ohbaboh@korea.kr (S.-D.O.); jyejin@korea.kr (Y.J.); 3 School of Applied Biosciences, Kyungpook National University, Daegu 41566, Korea; sjsuh@knu.ac.kr (S.-J.S.); psk@knu.ac.kr (S.-K.P.); 4 Department of Genetic Engineering and Graduate School of Biotechnology, Kyung Hee University, Yongin 17104, Korea; sunhwa@khu.ac.kr; 5 Department of Crop Science and Department of Smart Agriculture Systems, Chungnam National University, 99 Daehak-ro, Yuseong-gu, Daejeon 34134, Korea

**Keywords:** metabolic profiling, metabolite analysis, hybrid soybean, β-carotene-enhanced soybean, wild soybean

## Abstract

We obtained a new hybrid soybean (Hybrid) by hybridizing β-carotene-enhanced soybean (BCE; *Glycine max* L.) containing the *phytoene synthase-2A-carotene desaturase* gene and wild-type soybean (Wild; *Glycine soja*). To investigate metabolic changes between variants, we performed metabolic profiling of leaves (three growth stages) and seeds. Multivariate analyses revealed significant metabolic differences between genotypes in seeds and leaves, with seeds showing accumulation of phytosterols, tocopherols, and carotenoids (BCE only), indicating co-induction of the methylerythritol 4-phosphate and mevalonic acid pathways. Additionally, Hybrid produced intermediate levels of carotenoids and high levels of amino acids. Principal component analysis revealed metabolic discrimination between growth stages of soybean leaves and identified differences in leaf groups according to different genotypes at 8, 12, and 16 weeks, with Wild showing higher levels of environmental stress-related compounds relative to BCE and Hybrid leaves. The metabolic profiling approach could be a useful tool to identify metabolic links in various soybean cultivars.

## 1. Introduction

Soybean is consumed worldwide and represents an important crop grown for animal and human consumption as a high-quality protein source [1,2]. Soybean contains a variety of bioactive phytochemicals that provide beneficial effects to the human body [3]. For example, soybean contains isoflavones, tocopherols, soyasaponins, proteins, and oligosaccharides that are effective in various biological activities, such as cholesterol reduction, antioxidant activity, anticancer effects, and reductions in triglyceride levels [4,5,6,7,8,9]. Additionally, soybean leaf is closely associated with the development of soybean seed by supplying carbon sources to the seeds. The carbohydrate level of growing soybean leaf is regulated by interactions between carbohydrate production via photosynthesis, seed development, and other carbon sinks [10]. Thus, the composition of metabolites in leaves influences seed development.

Soybean is classified into *Glycine max* and *Glycine soja* species according to their habitat [11]. *Glycine max* is a cultivated soybean that is particularly rich in amino acids and used extensively in breeding and genetic modification to improve soybean production and nutritional value [11,12,13]. In contrast, *Glycine soja* is a wild species with greater genetic diversity and allele abundance than *Glycine max*. Additionally, previous studies found that *Glycine soja* exhibits better tolerance and adaptability to environmental stress, such as low temperature, dehydration, and salt stress, and high levels of phenylpropanoids and isoflavones [13,14,15,16,17]. Thus, *Glycine soja* is considered a gene source for improving the tolerance of cultivated species to biotic and abiotic stress [15,17]. Therefore, hybridization of *Glycine max* and *Glycine soja* could promote the development of new cultivated varieties with improved stress tolerance [18].

Carotenoids are a dietary precursor of vitamin A, a compound nutritionally beneficial to humans [19,20]. Because humans and animals cannot synthesize carotenoids in vivo, they obtain carotenoids by ingesting them through food. Carotenoids, such as β-carotene, α-carotene, and β-cryptoxanthin, are excellent antioxidants and effective in anti-aging and immunity, including reducing the risk of coronary artery disease, preventing cataracts and molecular degeneration, and preventing and treating some cancers and skin damage caused by ultraviolet rays [20,21]. Kim et al. [19] and Qin et al. [20] developed carotenoid-biofortified transgenic soybeans by inserting the *phytoene synthase-2A-carotene desaturase* (*PAC*) gene into *Glycine max* L. “Kwangan”, followed by nutritional-composition analysis. Additionally, previous studies have shown that hybridization of carotenoid-biofortified transgenic soybeans with improved nutritional value and wild soybeans promoted the production of soybeans with new nutritional value [22,23]. Continued research on hybrid soybeans between carotenoid-biofortified transgenic soybeans and wild soybeans is necessary to help develop new breeds based on the characteristics attainable by genetic hybridization.

Metabolomics is applied in conjunction with genomics and proteomics to gain a comprehensive understanding of complex networks of biological processes [24,25]. Because metabolites are the end products of gene expression and represent fundamental characteristics of the phenotype of an organism, metabolite levels depend on environmental and genetic changes. Thus, metabolomics research enables a deeper understanding of endogenous metabolic properties by comprehensively profiling and quantifying metabolites according to various genotypes [20]. Previous metabolomics studies of soybeans compared metabolic properties of genetically modified (GM) and non-GM soybeans or metabolic profiling of cultivars and wild species under various conditions, such as drying, high salt, and low temperature [14,16,26,27,28,29]. Although metabolomics research can be applied to compare metabolic differences under various conditions, comprehensive metabolic profiling of transgenic soybeans, wild soybeans, and their hybrid (*Glycine max* × *Glycine soja*) has not been performed.

In this study, we performed metabolic profiling to understand the metabolic properties of β-carotene-enhanced soybeans (BCE), wild soybeans (Wild), and hybrid soybeans (Hybrid). We applied gas chromatography–time-of-flight mass spectrometry (GC-TOF-MS), GC-quadrupole MS (GC-qMS), and high-performance liquid chromatography (HPLC) to identify primary and secondary metabolites in the seeds and leaves of BCE, Wild and Hybrid.

## 2. Materials and Methods

### 2.1. Samples and Chemicals

BCE were developed through *Agrobacterium*-mediated transformation using the recombinant *PAC* gene in cultivated soybean (*Glycine max* L. ‘Kwangan’) [19,30,31]. PI483463 was used as Wild, and Hybrid was generated from the hybridization of BCE and Wild [30,31]. Samples were provided by the Rural Development Administration (Jeonju, Korea). Seeds and leaves in three different growth stages (8, 12, and 16 weeks) from BCE, Wild, and Hybrid were used for the analyses. Soybean seeds and leaves were lyophilized in a freeze-dryer and then pulverized in a mortar and pestle. Soybean powders were kept in a freezer at −20 °C prior to analysis. Biological replicates were performed more than three times. Ribitol and 5α-cholestane, pyridine, *N*-methyl-*N*-(trimethylsilyl)trifluoroacetamide (MSTFA), and methoxyamine hydrochloride (MOX) were obtained from Sigma-Aldrich (St. Louis, MO, USA). All chemicals and reagents used in the study were HPLC grade.

### 2.2. Extraction and Analysis of Hydrophilic Compounds

The extraction method for hydrophilic compounds, such as amino acids, organic acids, sugars, phenolic acids, and sugar alcohols, was followed as described previously [32]. Powdered soybean sample (10 mg) was extracted with 1 mL of methanol:water:chloroform (2.5:1:1, *v/v/v*) solution, followed by the addition of 60 μL of ribitol solution (0.2 mg/mL in methanol) as an internal standard (IS) to the tube. The mixture was then incubated at 37 °C and shaken at 1200 rpm for 30 min in a thermomixer (model 5355; Eppendorf AG, Hamburg, Germany), followed by centrifugation at 16,000× *g* and 4 °C for 5 min. The supernatant (0.8 mL) was transferred to a new tube, and 0.4 mL of deionized water was added before centrifugation at 16,000× *g* and 4 °C for 5 min. The supernatant (0.9 mL) was then transferred to a new tube and dried in a centrifugal concentrator for at least 3 h, followed by freeze-drying for 16 h. For methoxime derivatization, 80 μL MOX (20 mg/mL) in pyridine was added and shaken at 30 °C for 90 min, after which 80 μL MSTFA was added and shaken for 30 min at 37 °C to perform trimethylsilylated etherification. These derivatized samples were analyzed using GC-TOF-MS. Hydrophilic compounds were analyzed on an Agilent 7890A GC (Agilent Technologies, Santa Clara, CA, USA) instrument equipped with a Pegasus TOF-MS (LECO, St. Joseph, MI, USA). The Rtx-5MS column (30 m × 0.25 mm, 0.25-μm i.d. film thickness; Restek, Bellefonte, PA, USA) was added to the GC, and helium gas was passed at a rate of 1 mL/min. Thereafter, 1 μL of extracted sample was injected at a 1:25 ratio in split mode, and the inlet temperature was set at 230 °C. The oven temperature was programmed as follows: starting at 80 °C for 2 min, followed by ramping to 320 °C at 15 °C/min and holding at this temperature for 50 min. The ion-source and transfer-line temperatures were respectively set at 250 °C and 280 °C. Spectral data were scanned over an *m*/*z* mass range of 85 to 600. For quantitative and qualitative analysis, we identified metabolites as compounds as Level 1 in the four-step reporting system recommended by the Chemical Analysis Working Group (CAWG) of the Metabolomics Standards Initiative (MSI) [33]. Data were analyzed using ChromaTOF software (v.5.5; LECO), and peaks were identified based on mass spectral data by comparison with in-house libraries, NIST 11 (https://www.nist.gov/, accessed on 1 October 2021), and Wiley 9 (https://sciencesolutions.wiley.com/spectral-databases/, accessed on 1 October 2021). For quantification, the relative peak-area ratio of compounds to that of the IS was acquired based on the selected ions (Table S1). Representative GC-TOF-MS chromatograms of soybean seeds and leaves are shown in Figure S1.

### 2.3. Carotenoid Extraction and Analysis

The extraction method used for carotenoid analysis was followed as described previously [34]. Briefly, for carotenoids analysis, soybean samples (10 mg leaves, 100 mg seeds) and 3 mL of 0.1% ascorbic acid in ethanol (*w/v*) were added to a 15 mL tube and vortexed. The samples were then placed in a water bath at 85 °C for 5 min. The mixture was then saponified with 120 μL of 80% potassium hydroxide in water (*w/v*) in the 85 °C water bath for 10 min. After saponification, the samples were placed immediately on ice for 5 min to stop the reaction, after which 100 μL of 25 ppm trans-β-Apo-8′-carotenal (as an IS), water (1.5 mL), and hexane (1.5 mL) were added to the tube and vortexed. After centrifugation for 5 min at 4 °C and 1200× *g*, the hexane layer (supernatant) was transferred to a new tube, and extraction was repeated with hexane (1.5 mL). The hexane layer (~3 mL) was then concentrated using nitrogen gas and a vacuum concentrator (VS-802F; Visionbionex, Gyeonggi, Korea). After dissolving the concentrate in 250 μL of methanol:dichloromethane (1:1, *v/v*) solution, the sample extract was filtered through a 0.5 μm syringe filter into 2 mL autosampler vial and analyzed by HPLC. Carotenoids were separated on a YMC carotenoid HPLC column (250 × 4.6 mm, 3 μm i.d.; YMC Co., Kyoto, Japan) using the HPLC system (Agilent 1100 HPLC instrument; Agilent Technologies) equipped with a diode-array detector with the wavelength set at 450 nm. The column temperature was set to 40 °C. Solvent A comprised methanol:water (92:8, *v/v*) with 10 mM ammonium acetate, and solvent B comprised 100% methyl tert-butyl ether. The binary gradient elution system of solvent A–solvent B was as follows: 0 min, 90% A/10% B; 20 min, 83% A/17% B; 29 min, 75% A/25% B; 35 min, 30% A/70% B; 40 min, 30% A/70% B; 42 min, 25% A/75% B; 45 min, 90% A/10% B; and 55 min, 90% A/10% B. For quantification, a calibration curve was obtained using standard compounds and calculated as the ratio of the peak area of the standard compound to the peak area of the IS (Figure S2).

### 2.4. Extraction and Analysis of Policosanols, Tocopherols, and Sterols

The extraction method used for secondary lipophilic compounds, such as policosanols, tocopherols, and sterols, was followed as described previously [13]. Soybean samples (10 mg of leaves, 15 mg of seeds) were extracted with 3 mL of 0.1% ascorbic acid ethanol (*w/v*), followed by the addition of 50 μL of 5α-cholestane (10 μg/mL) as an IS and incubation in a water bath at 85 °C for 10 min. Saponification was then performed with 120 μL of 80% potassium hydroxide (*w/v*), and the mixture was placed in a water bath at 85 °C for 10 min, followed by placement on ice for 5 min. The mixtures were then supplemented with hexane (1.5 mL) and water (1.5 mL) and centrifuged at 4 °C and 1200× *g* for 5 min, after which the supernatants were transferred to a new tube, and extraction was repeated with 1.5 mL hexane. The hexane (~3 mL) was dried using nitrogen gas and a vacuum concentrator. For derivatization, 30 μL MSTFA and 30 μL pyridine were added and incubated at 60 °C and 1200 rpm for 30 min, followed by analysis by GC-qMS using a GCMS-QP2010 Ultra system (Shimadzu, Kyoto, Japan). For separation, 1 µL aliquots were injected onto an Rtx-5MS column (30 m × 0.25 mm, 0.25 µm i.d.; Restek) at a split ratio of 1:10. Helium was flowed at a constant rate of 1 mL/min at an inlet temperature of 290 °C. The initial oven temperature (150 °C) was maintained for 2 min, ramped to 320 °C at 15 °C/min, and held for 10 min. The ion-source and interface temperatures were 230 °C and 280 °C, respectively. Spectra were acquired for *m/z* 85 to 600, and ions were detected in the selected ion-monitoring mode for peak analysis (Figure S3). Chromatographic data were processed using Labsolutions GCMS solution software (v.4.11; Shimadzu). For absolute quantification, accurate calibration curves were determined for each lipophilic standard at loadings of 0.025 µg to 5.00 µg and fixed to an IS weight of 0.50 µg (Table S2).

### 2.5. Statistical Analysis

All analyses were performed using at least three replicates. The relative quantification data and the absolute quantification data acquired from GC-TOF-MS, GC-qMS, and HPLC were scaled with unit variance scaling before all variables were subjected to the multivariate analysis. Principal component analysis (PCA) was performed using Soft Independent Modeling of Class Analogy software (v.13.0; Umetrics, Umeå, Sweden). Pearson’s correlation analysis was performed using the SAS software package (v.9.4; SAS Institute, Cary, NC, USA) to identify correlations between metabolites. Data analyzed in SAS were imported into MultiExperiment Viewer software (v.4.9.0; J. Craig Venter Institute, Rockville, MD, USA) for visualization and generation of hierarchical cluster analysis (HCA) results. The biological pathway was drawn based on the AtMetExpress overview pathway of *Arabidopsis thaliana* in WikiPathways (https://www.wikipathways.org/, accessed on 1 October 2021). PathVisio version 3.3.0 was downloaded from the PathVisio website (https://pathvisio.github.io/, accessed on 1 October 2021) and used for visualization of changes in metabolite. A *p* value < 0.05 was considered statistically significant.

## 3. Results and Discussion

### 3.1. PCA of Seeds

**Figure 1:**
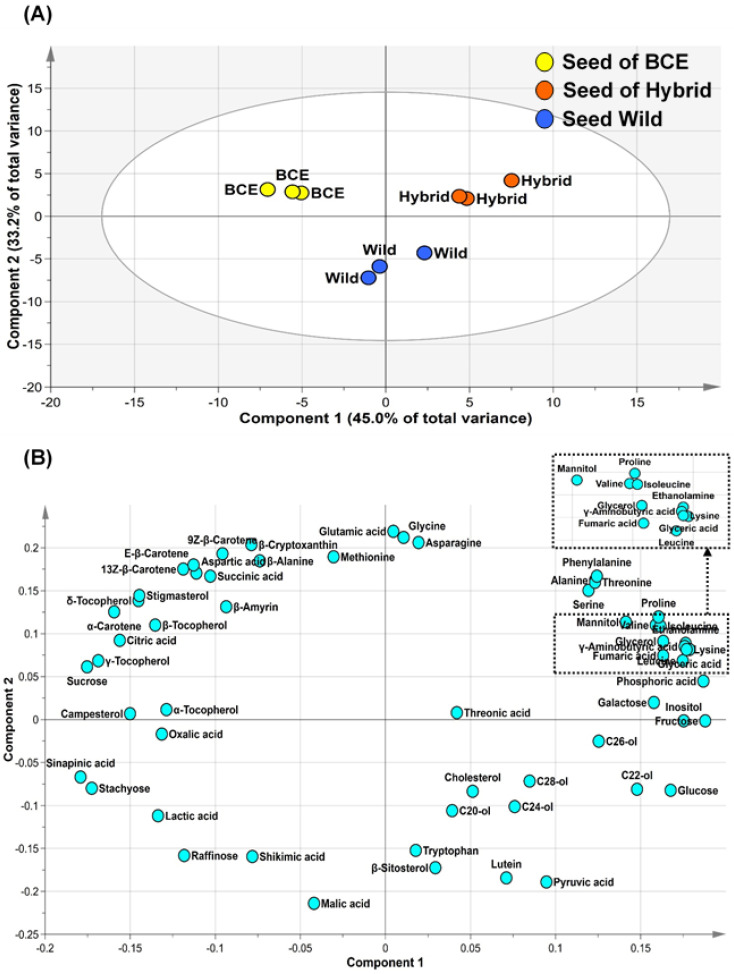
Score (**A**) and loading (**B**) plots of PCA of metabolites extracted from BCE, Hybrid, and Wild seeds.

We analyzed the primary metabolites (amino acids, organic acids, sugars, and sugar alcohols) and secondary metabolites (policosanols, phytosterols, carotenoids, and tocopherols) in seeds of BCE, Wild, and Hybrid using GC-TOF-MS, GC-qMS, and HPLC and obtained a total of 59 metabolites (Tables S3–S6). Analytical details for quantitative and qualitative analysis are given in Section 2.2, Section 2.3, Section 2.4 and Section 2.5, Tables S1 and S2. PCA is a step in multivariate analysis that can distinguish classes in highly-complex datasets [35]. We performed PCA to compare differences in metabolite composition among BCE, Hybrid, and Wild seeds with different genotypes, finding that two principal components explained 78.2% of the total variance in the data (Figure 1A). Principal component 1 (PC1) separated Hybrid and BCE seeds, and PC2 separated Wild seeds from other genotype seeds. To investigate the metabolites contributing to the separation among groups, we generated a loading plot (Figure 1B). Monosaccharides and most amino acids, except for aspartic acid, β-alanine, and methionine, showed clustered positive values for PC1. In contrast, sucrose, carotenoids (except for lutein), tocopherols, and polysaccharide showed negative values for PC1. These results showed that metabolites with positive values in PC1 were present in higher levels in Hybrid seed, whereas those with negative values were present in higher values in BCE seed. For PC2, policosanols, tryptophan, and organic acids (e.g., pyruvic acid, malic acid, lactic acid, shikimic acid, and sinapinic acid) presented negative values, with these metabolites presenting higher levels in Wild seed. The elevated content of shikimic acid was notable, as it represents a metabolite related to the phenylpropanoid biosynthesis pathway. A previous study reported that *Glycine soja* has high levels of flavonoids and phenylpropanoids [13]. 

### 3.2. Pearson’s Correlation Analysis and HCA of Seeds

**Figure 2:**
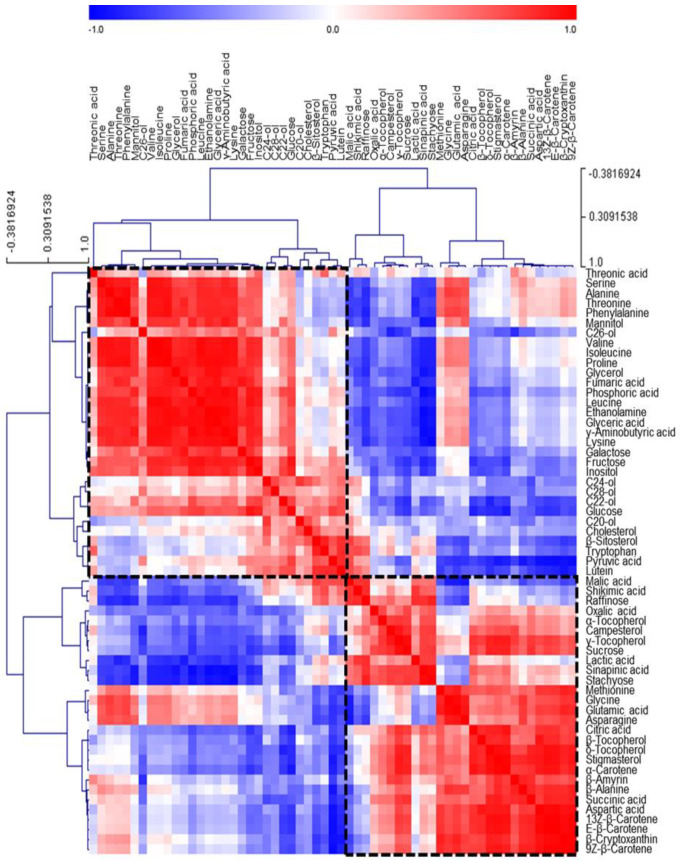
Correlation matrix and cluster analysis of data for 59 metabolites of soybean seeds. Each square indicates the Pearson’s correlation coefficient of a pair of compounds, and the value of the correlation coefficient is represented by the intensity of blue or red colors, as indicated on the color scale. Hierarchical clusters are presented as a cluster tree. C20-ol, eicosanol; C22-ol, docosanol; C24-ol, tetracosanol; C26-ol, hexacosanol; C28-ol, octacosanol.

We then performed Pearson’s correlation analysis and HCA to investigate relationships between the 59 metabolites identified in the three soybean seeds (Figure 2). HCA results between metabolites revealed that most amino acids, monosaccharides, policosanol, pyruvic acid, and lutein were grouped in cluster 1, whereas TCA cycle intermediates (except fumaric acid), polysaccharides, sucrose, tocopherols, most phytosterols, and carotenoids (except lutein) were grouped in cluster 2. Thus, cluster 1 mainly comprised nitrogen compounds, such as amino acids, whereas most of the carbon compounds, including isoprenoids and organic acids related to the TCA cycle, were included in cluster 2. Moreover, metabolites related to closely-linked pathways clustered together, showing high correlation. Furthermore, carotenoids, tocopherols, and phytosterols, such as stigmasterol, campesterol, and β-amyrin clustered in cluster 2 demonstrated positive correlations. These results provided insight into relationships between metabolites associated with the isoprenoid pathways. Energy metabolism in plants regulates various pathways, including photosynthesis and amino acid biosynthesis, and closely controls carbon and nitrogen metabolism for growth via extensive interactions [36]. The results of the present study offer insight into the complex interactions between carbon and nitrogen metabolites related to sugar metabolism, the TCA cycle, and amino acid metabolism in plants. Monosaccharides, including glucose, galactose, and fructose, showed negative correlations with polysaccharides and disaccharides, such as raffinose, stachyose, and sucrose. Because polysaccharides are hydrolyzed to monosaccharides to produce energy for seed growth, monosaccharide levels appear high at the stage of vigorous growth [29]. As most seeds mature, polysaccharide levels increase, whereas monosaccharide levels decrease [37].

### 3.3. Pathway Analysis of Seeds

**Figure 3:**
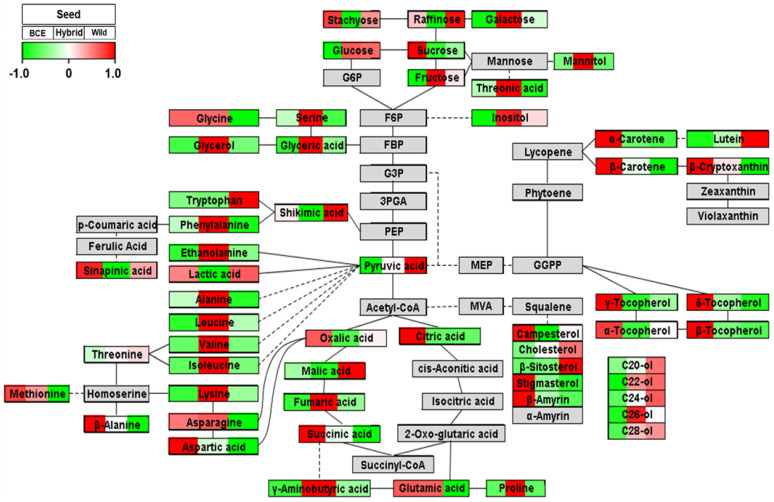
Metabolic pathways visualization of BCE, Hybrid, and Wild seeds. The UV scaling value range is from −1.0 to 1.0. A UV scaling value >0 indicates higher levels than average in each value of BCE, Hybrid, and Wild (shown in red). A UV scaling value <0 indicates lower than average values and is shown in green. Gray color represents an undetected compound.

PCA and HCA results identified differences in metabolite patterns in BCE, Wild, and Hybrid seeds, with these differences visualized as metabolic pathways (Figure 3) according to the average UV scaling values (rang: −1.0 to 1.0). Analytical results of the isoprenoid pathway showed significant differences between BCE, Hybrid, and Wild seeds. Carotenoids were the main metabolites induced by introduction of the *phytoene synthase* and *carotene desaturase* genes, with total carotenoid content at 255.8 μg/g, 88.7 μg/g, and 8.2 μg/g in BCE, Hybrid, and Wild, respectively (Table S6). Therefore, the results confirmed the effects intended by genetic transformation and hybridization. The contents of tocopherols and phytosterols (β-amyrin, campesterol, and stigmasterol) were highest in BCE seed and no difference was observed between Hybrid and Wild seeds. Thus, the results demonstrated that non-mevalonate and mevalonate pathways were activated in BCE seeds by inserting the *PAC* gene as an unexpected effect. Carotenoids, tocopherols and phytosterols are well known as antioxidants [3,17,38]. Additionally, BCE seeds showed lower levels of pyruvic acid than Wild and Hybrid seeds. The first step in carotenoid biosynthesis requires processing of pyruvic acid via a non-mevalonate pathway [39]. Moreover, HCA results showed negative correlations between pyruvic acid and carotenoid levels (Figure 2). For example, pyruvic acid was significantly (*p* < 0.0002) negatively correlated with 9Z-β-carotene (*r* = −0.95698), E-β-carotene (*r* = −0.95513), and 13Z-β-carotene (*r* = −0.94891), suggesting that pyruvic acid was used as a precursor for the production of carotenoids and resulting in lower levels of pyruvic acid in BCE seeds with higher levels of carotenoids than Hybrid and Wild seeds.

Phenylpropanoid is a defense compound induced by a variety of biotic and abiotic stresses, such as pathogen attack, wounding, and ozone and ultraviolet light exposure, and serves as a precursor to polyphenol-barrier synthesis [40]. Tryptophan and phenylalanine are an essential amino acid for human and are precursors for flavonoid biosynthesis pathways [40]. Phenylalanine levels were lower in Wild seed than in the other seeds, indicating that phenylalanine was used as a precursor for the production of phenolic acid. Previous studies have reported that wild-type soybeans show relatively high levels of phenylpropanoids and isoflavones than cultivated soybeans [13,17]. Additionally, levels of policosanols were higher in Wild seed relative to BCE and Hybrid seeds. Policosanol is an aliphatic primary alcohol and a protective component present in the entire seed and wax layer [41].

Furthermore, levels of most amino acids were significantly higher in Hybrid seed than in BCE and Wild seeds. Oh et al. [31] reported that the content of crude protein in Hybrid seed was higher than that in BCE and Wild seeds. 

### 3.4. PCA of Leaves

**Figure 4:**
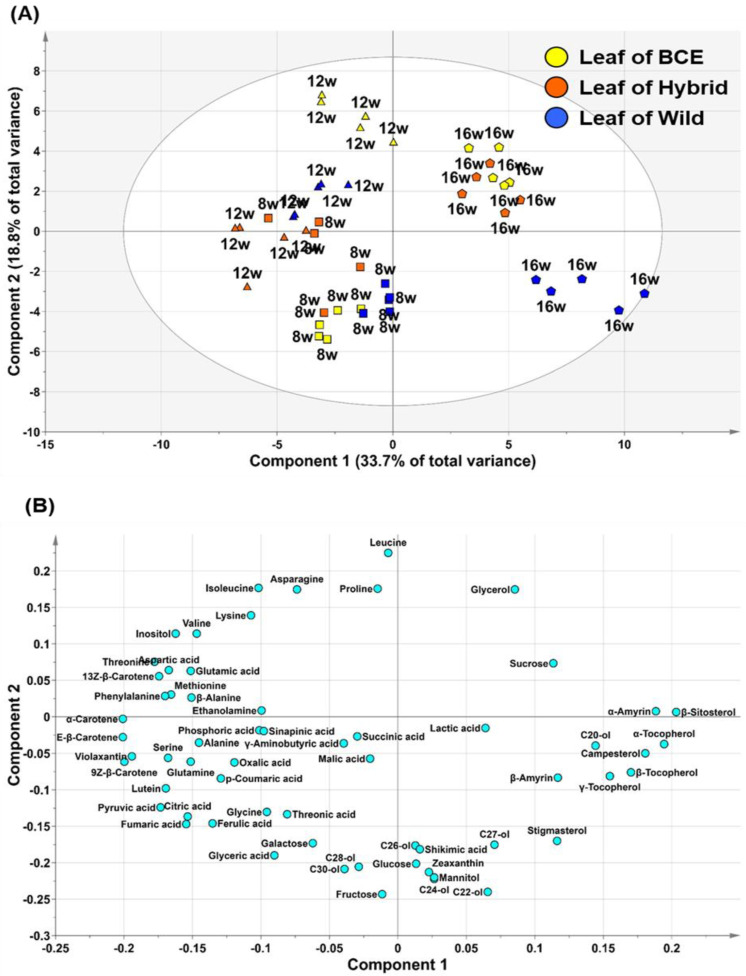
Score (**A**) and loading (**B**) plots of PCA of metabolites extracted from BCE, Hybrid, and Wild leaves. Squares, triangles, and pentagons represent 8, 12, and 16 weeks, respectively.

Soybean leaves store energy through photosynthesis and transfer it to storage organs, such as seeds. Given that leaves are closely related to seeds, we performed metabolite profiling of leaves together with the seeds. We analyzed a total of 61 hydrophilic and lipophilic components in the leaves of the soybeans (Tables S7–S10, see Section 2.2, Section 2.3, Section 2.4 and Section 2.5, Tables S1 and S2 for more details) and performed PCA to confirm differences in the composition of metabolites according to genotype and growth stage (leaves of BCE, Wild, and Hybrid leaves at 8, 12, and 16 weeks). The PCA results were separated according to growth stage rather than leaf genotype (Figure 4). The two principal components, accounting for 52.5% of the total variance, separated the leaves of all genotype soybeans according to their growth stage. In PC1, sucrose, tocopherols, amyrins, and sterols were grouped with positive values. In contrast, most carotenoids, organic acids, monosaccharides, and amino acids presented negative values in PC1. These results showed that in leaves, levels of most primary metabolites and carotenoids decreased, whereas levels of secondary metabolites (plant sterols and tocopherols) increased along with growth in all genotype soybeans, which was consistent with previous studies [29,42,43]. Plants store large amounts of energy through photosynthesis, and carotenoids are an essential compound involved in photosynthesis [39]. Young leaves fix large amounts of carbon through photosynthesis, and the fixed carbon remains in the form of monosaccharides and is used as an energy source for plant growth or transfer of energy to other tissues or organs in the form of glucose [44]. In contrast, in mature leaves, organic acids, such as citric acid and succinic acid, are either oxidized through the TCA cycle or synthesized into sucrose through gluconeogenesis and transported to other tissues [45,46]. The secondary metabolites phytosterol and tocopherol are considered representative antioxidants produced in response to oxidative stress [38,41,47]. Furthermore, phytosterols are a major structural component of biological membranes and involved in plant responses to biotic and abiotic stress [39]. The present study demonstrated that 16-week-old leaves produced more antioxidants and more defenses against aging or stress. In particular, wild leaves at 16 weeks (*Glycine soja*) showed higher levels of shikimic acid, γ-aminobutyric acid, policosanols, and phenolic acids, such as sinapinic acid, *p*-coumaric acid, and ferulic acid. Phenolic compounds are mainly present in the outer layers of plants to protect against environmental stress, such as dryness and salt [41,48]. The findings of the present study suggest that the phenylpropanoid pathway in the Wild leaf is induced as a defense mechanism for survival. Similarly, γ-aminobutyric acid accumulates in response to biological and abiotic stress [43], and Ling et al. [49] reported that γ-aminobutyric acid contributes to the rigidity and extensibility of the cell wall. Therefore, the increase in secondary metabolites at maturity could be an important factor in determining the metabolic properties of aging in soybean plants. 

In addition, we performed PCA to compare leaves with different genotypes in the same growth stage. A biplot is a standard way to show sample scores and loadings in a single plot. All PCA results at the same growth stage (8, 12, and 16 weeks, respectively) showed that BCE, Wild, and Hybrid leaves were divided according to genotype (Figure S4). The present study showed that BCE leaves had higher levels of sucrose at the same growth stage than other genotype leaves, and that most amino acids and carotenoids were higher in BCE and hybrid leaves than wild leaves (Figure S4). In our results, sucrose levels were higher in BCE leaves than other genotype leaves at the same growth stage, and most amino acids and carotenoids were higher in BCE and hybrid leaves than in wild leaves (Figure S4). Previous studies show that leaves of *Glycine max* have higher levels of most amino acids and sucrose than leaves of *Glycine soja* [28,29]. This is because cultivated soybeans have been developed to accumulate more biomass than wild soybeans [50]. Therefore, higher levels of photosynthesis and energy metabolism are required in leaves of cultivated soybeans. We speculate that this is because more energy and nitrogen sources should be provided from leaves to seeds for activation of energy metabolism and amino acid metabolism in BCE and Hybrid seeds.

## 4. Conclusions

This study represents the first analysis of primary metabolites, including organic acids, amino acids, sugars, and sugar alcohols, and secondary metabolites, including carotenoids, tocopherols, phytosterols, policosanols, and phenolic acids, using GC-TOF-MS, HPLC, and GC-qMS to investigate the metabolic patterns of BCE, Wild, and Hybrid induced by their hybridization. Metabolic profiling has been employed to direct breeding strategies to enhance specific desired balances of food components in fresh food [13]. Multivariate and metabolic pathway analyses revealed that soybean seeds and leaves of the three different genotypes demonstrated different metabolic patterns. Consumers are aware of the need for a constant supply of phytochemical-containing foods for antioxidant support and disease prevention [34]. BCE seeds exhibited high levels of carotenoids, tocopherols, and three phytosterols that are effective in several biological activities, such as cholesterol reduction and antioxidant activity [5,19]. Hybrid seeds contained high levels of amino acids, and Wild seeds showed high levels of policosanols. Especially, Hybrid seeds had high γ-aminobutyric acid levels which has been shown to play a role in neurological function, such as in Alzheimer’s disease, epilepsy, and anxiety disorders [51]. Additionally, soybean leaves of all genotypes presented decreases in primary metabolites and increases in secondary metabolites during growth, although we observed differences in metabolite composition between genotypes at the same growth stage. Therefore, these results demonstrated differences in metabolic profiles in seeds and leaves of BCE, Wild and Hybrid. Thus, targeted metabolite profiling is proposed as an appropriate tool for metabolic phenotyping of soybean seeds and it should provide useful information for developing nutrition-rich soybean cultivars. Furthermore, future work should involve a more hybrid soybean sample to develop soybean-based products through better understanding of the metabolic phenotypes in the breeding of new cultivars. 

## Data Availability

The data presented in this study are contained within the article or supplementary material.

## Supplementary Materials

The following are available online at https://www.mdpi.com/article/10.3390/foods10102421/s1, Figure S1: GC-TOF-MS analytical ion chromatogram (AIC) of hydrophilic compounds extracted from BCE seeds (A) and leaves (B). 1, Pyruvic acid; 2, Lactic acid; 3, Alanine; 4, Oxalic acid; 5, Valine; 6, Urea; 7, Serine-1; 8, Glycerol; 9, Ethanolamine; 10, Leucine; 11, Phosphoric acid; 12, Isoleucine; 13, Proline; 14, Succinic acid; 15, Glycine; 16, Glyceric acid; 17, Fumaric acid; 18, Serine-2; 19, Threonine; 20, β-Alanine; 21, Malic acid; 22, Aspartic acid; 23, Methionine; 24, γ-Aminobutyric acid; 25, Threonic acid; 26, Glutamic acid; 27, Phenylalanine; 28, Xylose; 29, Arabinose; 30, Asparagine; 31, Xylitol; 32, Ribitol; 33, Glutamine; 34, Shikimic acid; 35, Citric acid; 36, Quinic acid; 37, Fructose-1; 38, Fructose-2; 39, Mannose; 40, Galactose-1; 41, Glucose-1; 42, Lysine; 43, Ferulic acid; 44, Glucose-2; 45, Mannitol; 46, *p*-Coumaric acid; 47, Sinapinic acid; 48, Inositol; 49, Tryptophane; 50, Sucrose; 51, Raffinose; 52, Stachyose, Figure S2: HPLC chromatogram of carotenoid compounds from seeds (A) and leaves (B). Peak: 1, Violaxanthin; 2, Lutein; 3, Zeaxanthin; 4, trans-β-Apo-8′-carotenal (IS); 5, β-Cryptoxanthin; 6, 13Z-β-Carotene; 7, α-Carotene; 8, E-β-Carotene; 9, 9Z-β-Carotene, Figure S3: GC-qMS total ion chromatogram (TIC) of seeds (A) and leaves (B). 1, C20-ol; 2, C22-ol; 3, C24-ol; 4, δ-Tocopherol; 5, 5α-Cholestane; 6, C26-ol; 7, β-Tocopherol; 8, γ-Tocopherol; 9, C27-ol; 10, C28-ol; 11, α-Tocopherol; 12, Cholesterol; 13, Campesterol; 14, C30-ol; 15, Stigmasterol; 16, β-Sitosterol; 17, β-Amyrin; 18, α-Amyrin. Figure S4. Biplots of PCA of metabolites extracted from BCE, Hybrid, and Wild leaves at 8 weeks (A), 12 weeks (B), and 16 weeks (C). Circles and triangles in the biplot represent samples and metabolites, respectively. Table S1. Relative retention times (RRT) and mass spectrometry data of hydrophilic compounds in soybean leaf and seed. Table S2. Relative retention times (RRT) and mass spectrometry data of lipophilic compounds in soybean leaf and seed. Table S3. Raw data of hydrophilic compounds of soybean seeds identified using GC-TOF-MS. Table S4. Raw data of lipophilic compounds of soybean seeds identified using GC-MS and HPLC. Table S5. Composition and content (ratio/g) of hydrophilic compounds in soybean seeds with GC-TOF-MS analysis. Table S6. Composition and content (μg/g) of lipophilic compounds in soybean seeds with GC-MS and HPLC analysis. Table S7. Raw data of hydrophilic compounds of soybean leaves identified using GC-TOF-MS. Table S8. Raw data of lipophilic compounds of soybean leaves identified using GC-MS and HPLC. Table S9. Composition and content (ratio/g) of hydrophilic compounds in soybean leaves with GC-TOF-MS analysis. Table S10. Composition and content (μg/g) of lipophilic compounds in soybean leaves with GC-MS and HPLC analysis.
