# Supplementary material for: Metabolomic Variability of Different Soybean Genotypes: β-Carotene-Enhanced (Glycine max), Wild (Glycine soja), and Hybrid (Glycine max × Glycine soja) Soybeans
Source: Foods. 2021 Oct 13;10(10):2421. doi: 10.3390/foods10102421 (PMC8535314; doi:10.3390/foods10102421)
Supplement: Supplementary file 1 [file foods-10-02421-s001.zip › Foods-1366710 supplementary figure_revision_├╓┴╛.pptx]

## Slide 1
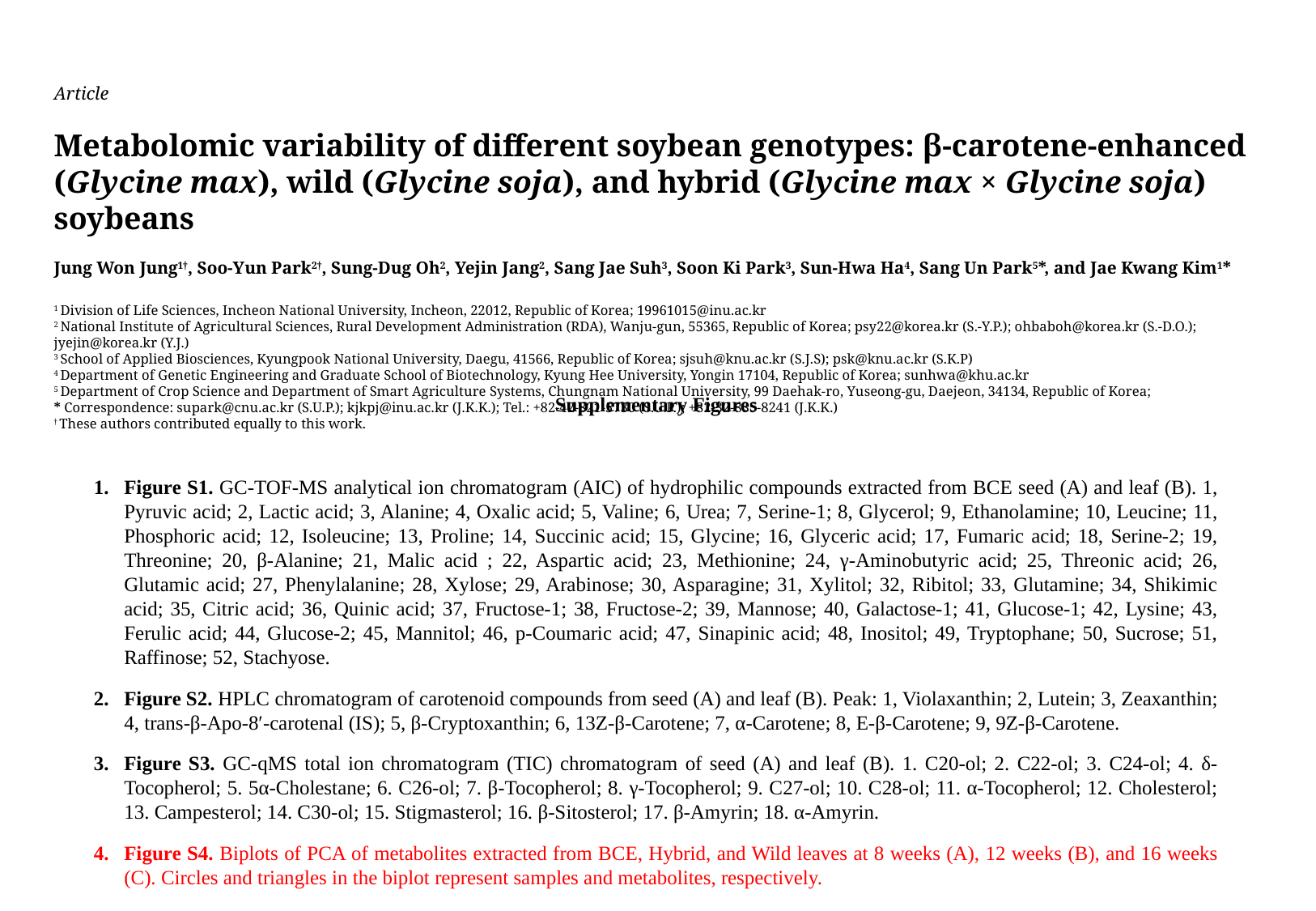

Article
Metabolomic variability of different soybean genotypes: β-carotene-enhanced (Glycine max), wild (Glycine soja), and hybrid (Glycine max × Glycine soja) soybeans
Jung Won Jung1†, Soo-Yun Park2†, Sung-Dug Oh2, Yejin Jang2, Sang Jae Suh3, Soon Ki Park3, Sun-Hwa Ha4, Sang Un Park5*, and Jae Kwang Kim1*
 
1 Division of Life Sciences, Incheon National University, Incheon, 22012, Republic of Korea; 19961015@inu.ac.kr
2 National Institute of Agricultural Sciences, Rural Development Administration (RDA), Wanju-gun, 55365, Republic of Korea; psy22@korea.kr (S.-Y.P.); ohbaboh@korea.kr (S.-D.O.); jyejin@korea.kr (Y.J.)
3 School of Applied Biosciences, Kyungpook National University, Daegu, 41566, Republic of Korea; sjsuh@knu.ac.kr (S.J.S); psk@knu.ac.kr (S.K.P)
4 Department of Genetic Engineering and Graduate School of Biotechnology, Kyung Hee University, Yongin 17104, Republic of Korea; sunhwa@khu.ac.kr
5 Department of Crop Science and Department of Smart Agriculture Systems, Chungnam National University, 99 Daehak-ro, Yuseong-gu, Daejeon, 34134, Republic of Korea;
* Correspondence: supark@cnu.ac.kr (S.U.P.); kjkpj@inu.ac.kr (J.K.K.); Tel.: +82-42-821-5730 (S.U.P.); +82-32-835-8241 (J.K.K.)
† These authors contributed equally to this work.
Supplementary Figures
Figure S1. GC-TOF-MS analytical ion chromatogram (AIC) of hydrophilic compounds extracted from BCE seed (A) and leaf (B). 1, Pyruvic acid; 2, Lactic acid; 3, Alanine; 4, Oxalic acid; 5, Valine; 6, Urea; 7, Serine-1; 8, Glycerol; 9, Ethanolamine; 10, Leucine; 11, Phosphoric acid; 12, Isoleucine; 13, Proline; 14, Succinic acid; 15, Glycine; 16, Glyceric acid; 17, Fumaric acid; 18, Serine-2; 19, Threonine; 20, β-Alanine; 21, Malic acid ; 22, Aspartic acid; 23, Methionine; 24, γ-Aminobutyric acid; 25, Threonic acid; 26, Glutamic acid; 27, Phenylalanine; 28, Xylose; 29, Arabinose; 30, Asparagine; 31, Xylitol; 32, Ribitol; 33, Glutamine; 34, Shikimic acid; 35, Citric acid; 36, Quinic acid; 37, Fructose-1; 38, Fructose-2; 39, Mannose; 40, Galactose-1; 41, Glucose-1; 42, Lysine; 43, Ferulic acid; 44, Glucose-2; 45, Mannitol; 46, p-Coumaric acid; 47, Sinapinic acid; 48, Inositol; 49, Tryptophane; 50, Sucrose; 51, Raffinose; 52, Stachyose.
Figure S2. HPLC chromatogram of carotenoid compounds from seed (A) and leaf (B). Peak: 1, Violaxanthin; 2, Lutein; 3, Zeaxanthin; 4, trans-β-Apo-8′-carotenal (IS); 5, β-Cryptoxanthin; 6, 13Z-β-Carotene; 7, α-Carotene; 8, E-β-Carotene; 9, 9Z-β-Carotene.
Figure S3. GC-qMS total ion chromatogram (TIC) chromatogram of seed (A) and leaf (B). 1. C20-ol; 2. C22-ol; 3. C24-ol; 4. δ-Tocopherol; 5. 5α-Cholestane; 6. C26-ol; 7. β-Tocopherol; 8. γ-Tocopherol; 9. C27-ol; 10. C28-ol; 11. α-Tocopherol; 12. Cholesterol; 13. Campesterol; 14. C30-ol; 15. Stigmasterol; 16. β-Sitosterol; 17. β-Amyrin; 18. α-Amyrin.
Figure S4. Biplots of PCA of metabolites extracted from BCE, Hybrid, and Wild leaves at 8 weeks (A), 12 weeks (B), and 16 weeks (C). Circles and triangles in the biplot represent samples and metabolites, respectively.

## Slide 2
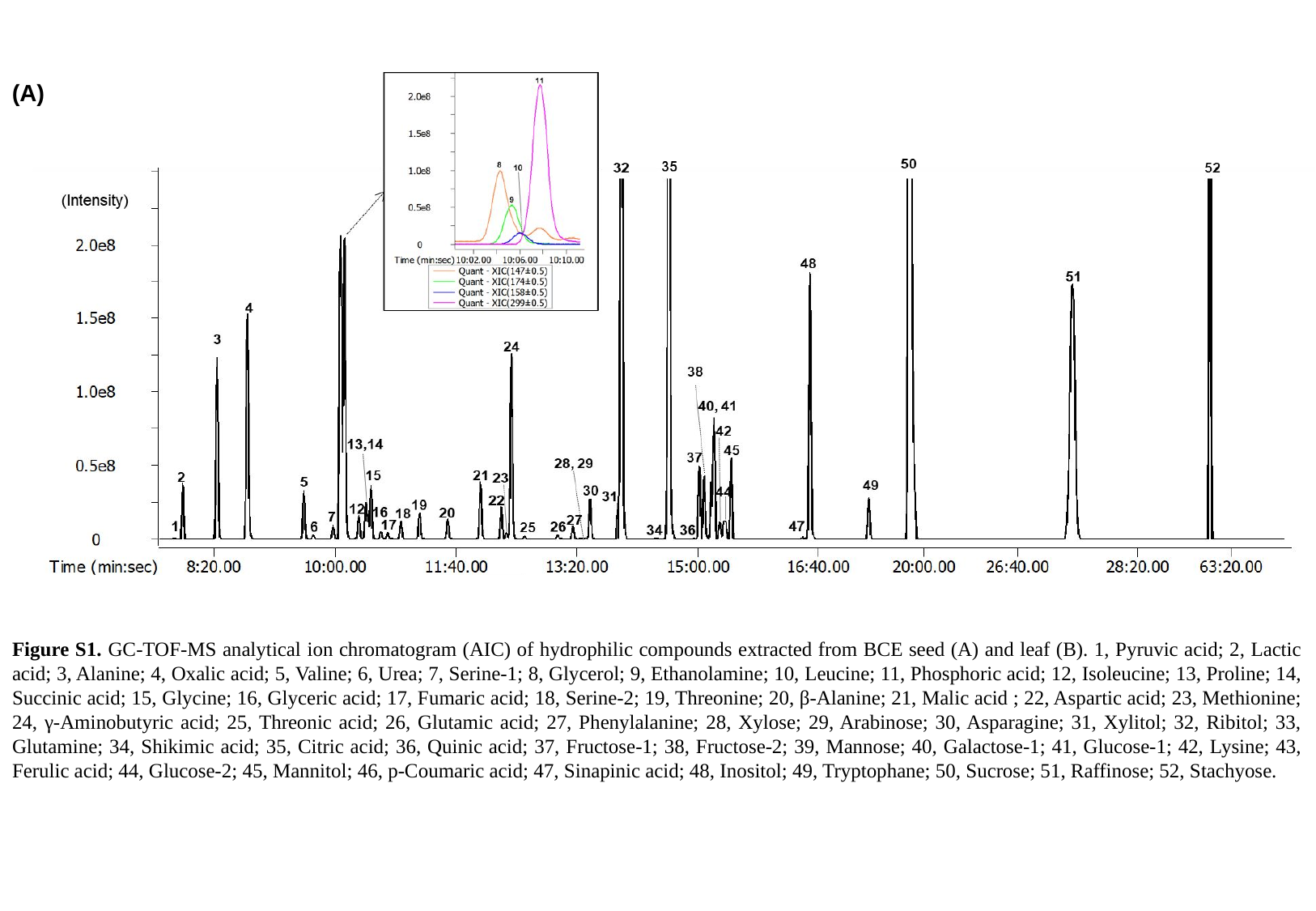

(A)
Figure S1. GC-TOF-MS analytical ion chromatogram (AIC) of hydrophilic compounds extracted from BCE seed (A) and leaf (B). 1, Pyruvic acid; 2, Lactic acid; 3, Alanine; 4, Oxalic acid; 5, Valine; 6, Urea; 7, Serine-1; 8, Glycerol; 9, Ethanolamine; 10, Leucine; 11, Phosphoric acid; 12, Isoleucine; 13, Proline; 14, Succinic acid; 15, Glycine; 16, Glyceric acid; 17, Fumaric acid; 18, Serine-2; 19, Threonine; 20, β-Alanine; 21, Malic acid ; 22, Aspartic acid; 23, Methionine; 24, γ-Aminobutyric acid; 25, Threonic acid; 26, Glutamic acid; 27, Phenylalanine; 28, Xylose; 29, Arabinose; 30, Asparagine; 31, Xylitol; 32, Ribitol; 33, Glutamine; 34, Shikimic acid; 35, Citric acid; 36, Quinic acid; 37, Fructose-1; 38, Fructose-2; 39, Mannose; 40, Galactose-1; 41, Glucose-1; 42, Lysine; 43, Ferulic acid; 44, Glucose-2; 45, Mannitol; 46, p-Coumaric acid; 47, Sinapinic acid; 48, Inositol; 49, Tryptophane; 50, Sucrose; 51, Raffinose; 52, Stachyose.

## Slide 3
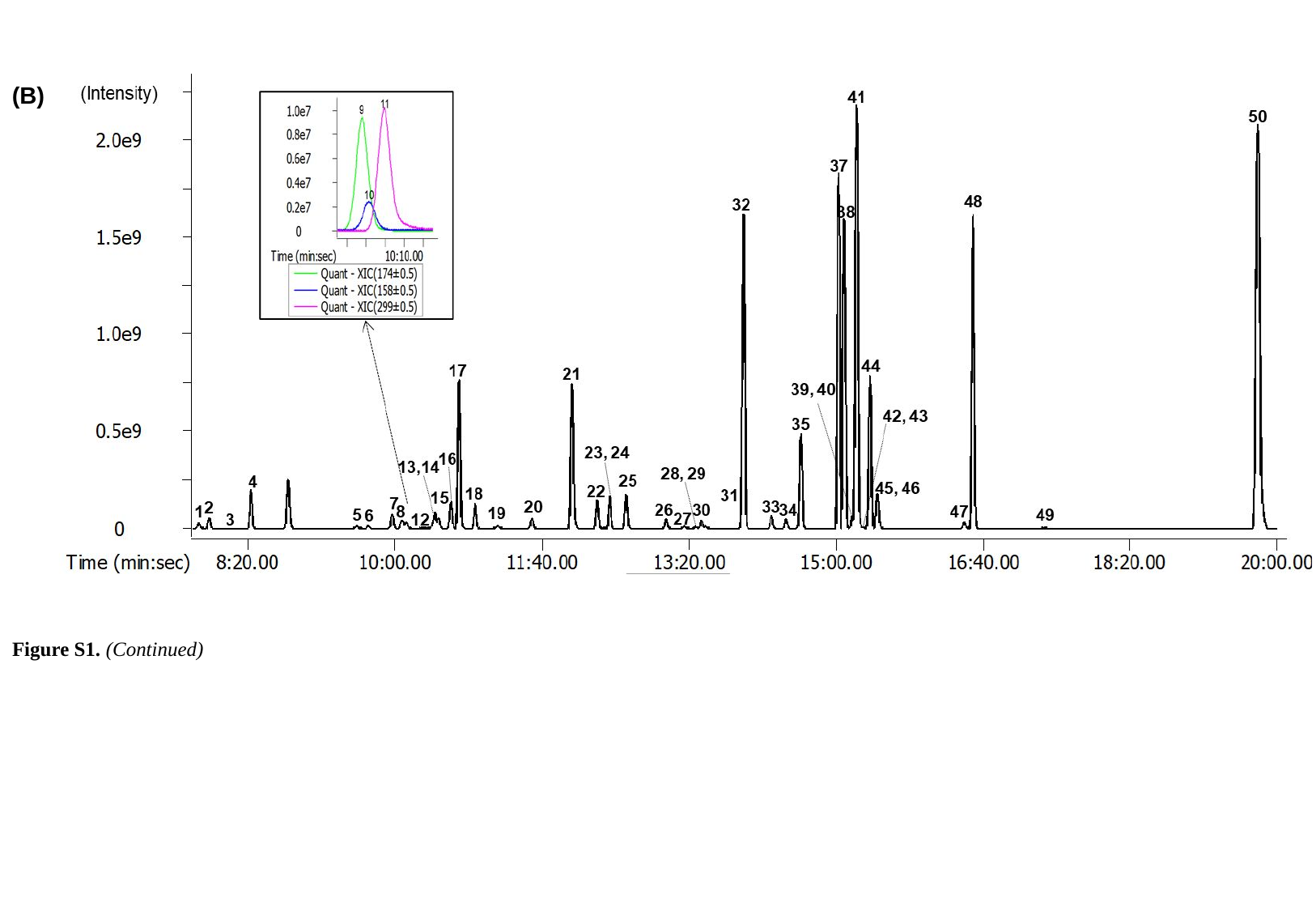

(B)
Figure S1. (Continued)

## Slide 4
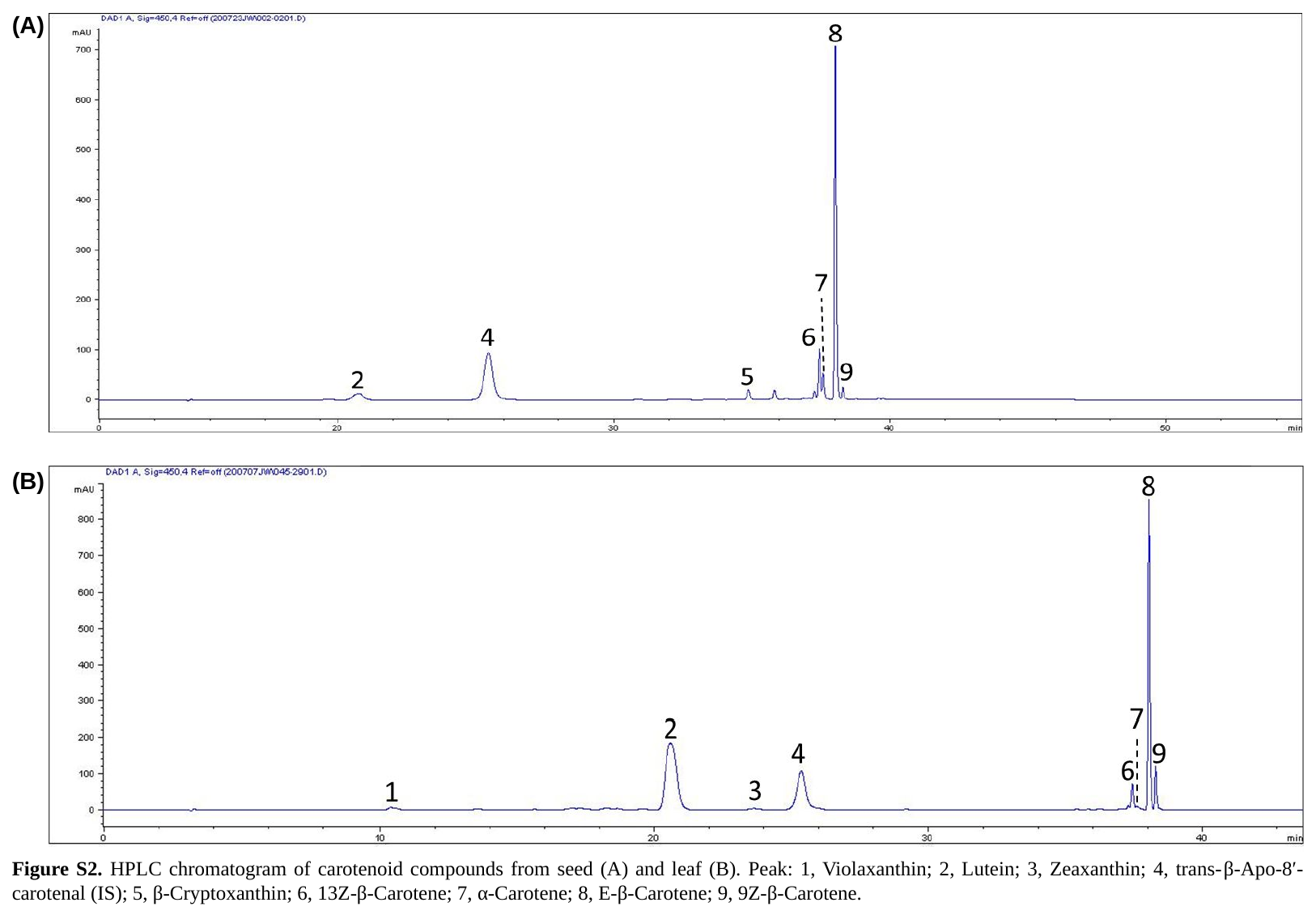

(A)
(B)
Figure S2. HPLC chromatogram of carotenoid compounds from seed (A) and leaf (B). Peak: 1, Violaxanthin; 2, Lutein; 3, Zeaxanthin; 4, trans-β-Apo-8′-carotenal (IS); 5, β-Cryptoxanthin; 6, 13Z-β-Carotene; 7, α-Carotene; 8, E-β-Carotene; 9, 9Z-β-Carotene.

## Slide 5
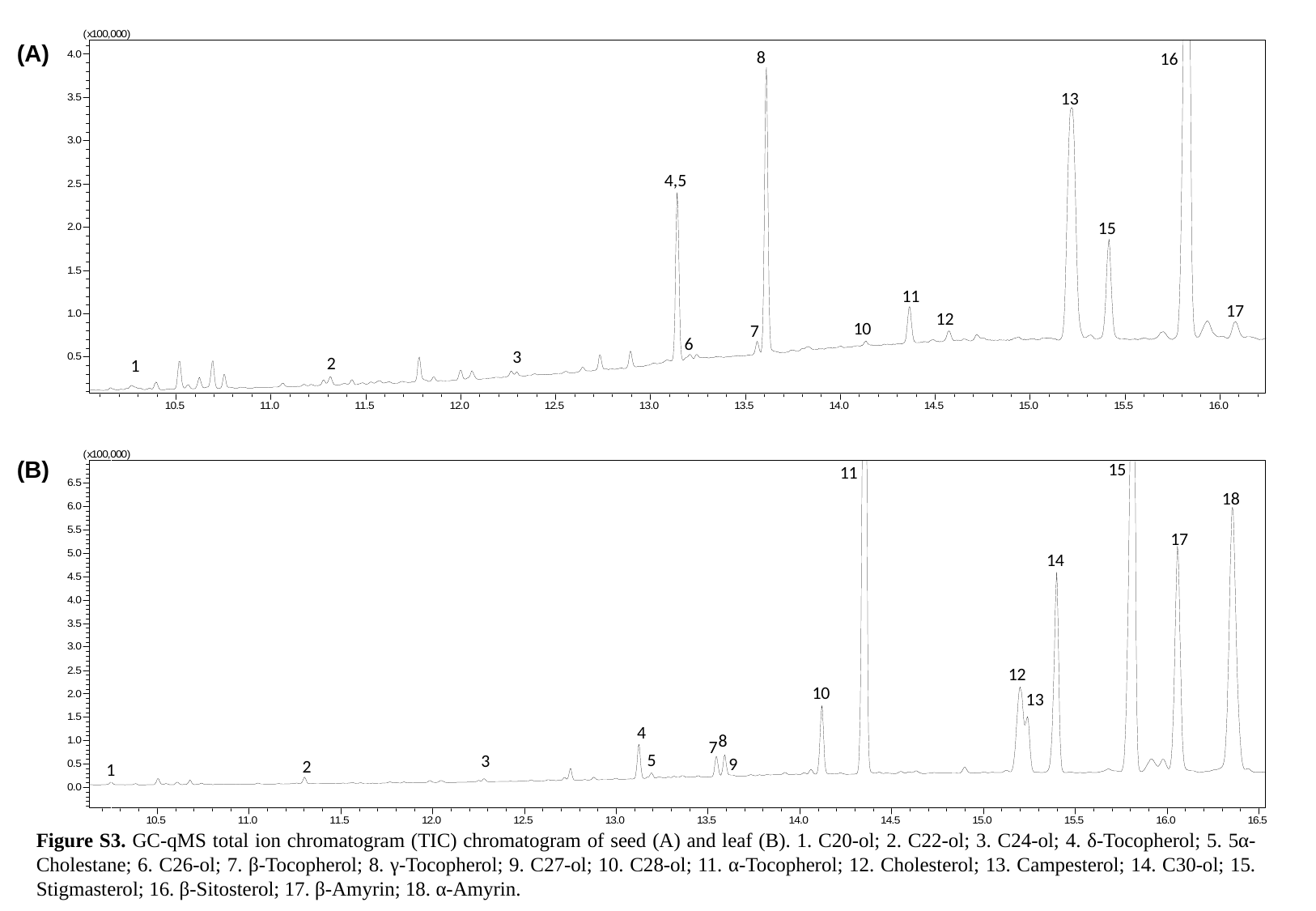

8
16
13
4,5
15
11
17
12
10
7
6
3
2
1
(A)
15
11
18
17
14
12
10
13
4
8
7
5
3
9
2
1
(B)
Figure S3. GC-qMS total ion chromatogram (TIC) chromatogram of seed (A) and leaf (B). 1. C20-ol; 2. C22-ol; 3. C24-ol; 4. δ-Tocopherol; 5. 5α-Cholestane; 6. C26-ol; 7. β-Tocopherol; 8. γ-Tocopherol; 9. C27-ol; 10. C28-ol; 11. α-Tocopherol; 12. Cholesterol; 13. Campesterol; 14. C30-ol; 15. Stigmasterol; 16. β-Sitosterol; 17. β-Amyrin; 18. α-Amyrin.

## Slide 6
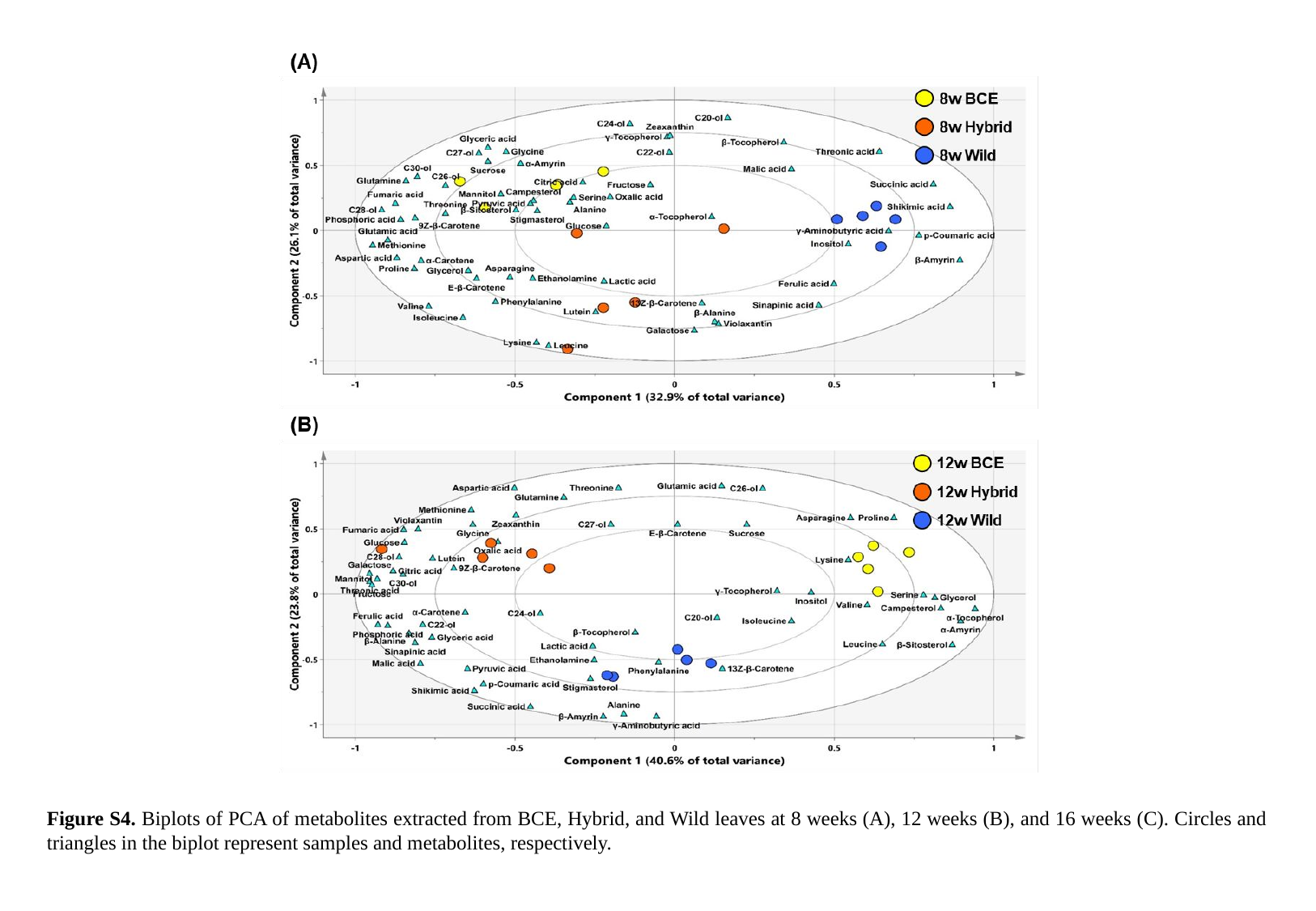

Figure S4. Biplots of PCA of metabolites extracted from BCE, Hybrid, and Wild leaves at 8 weeks (A), 12 weeks (B), and 16 weeks (C). Circles and triangles in the biplot represent samples and metabolites, respectively.

## Slide 7
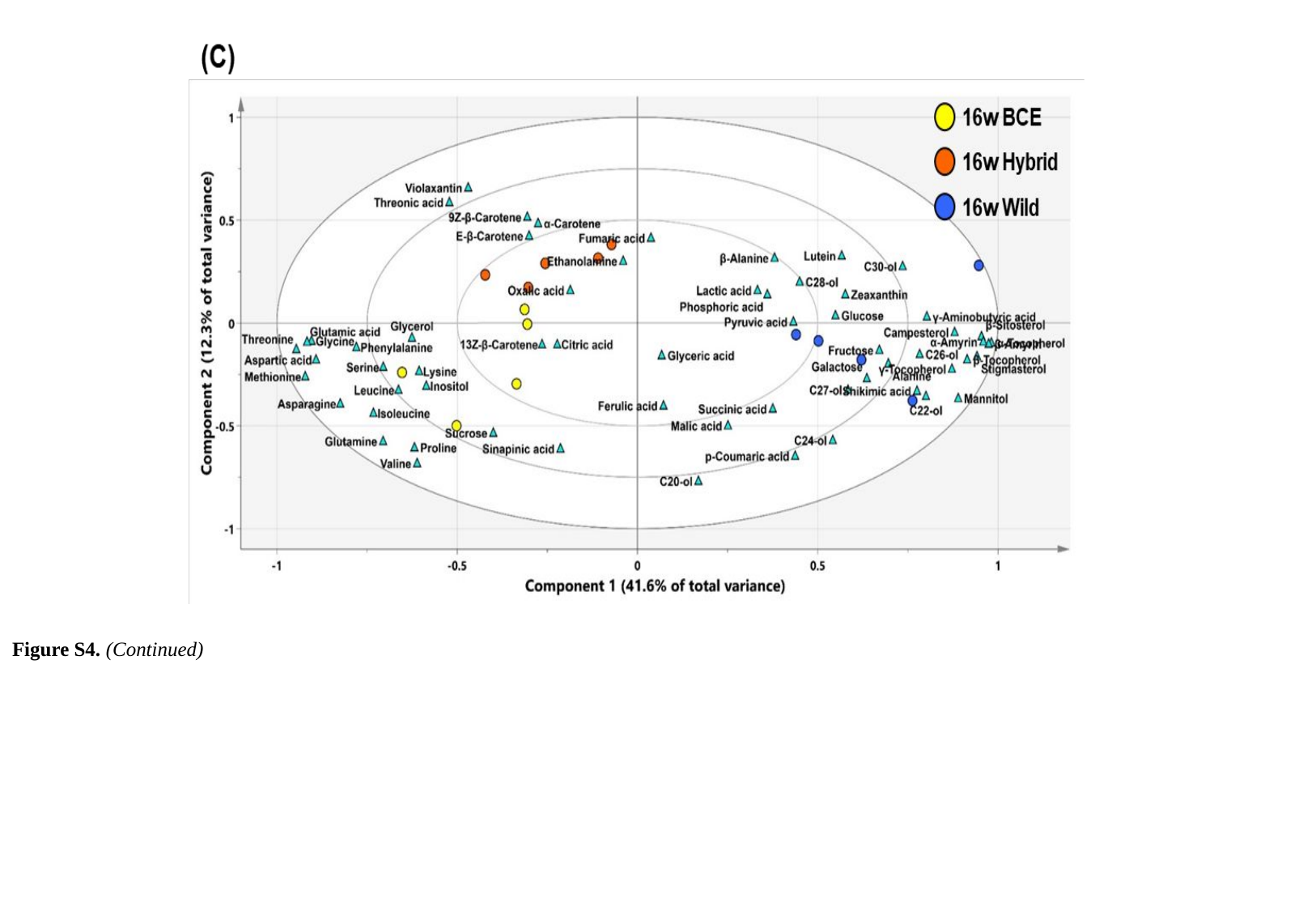

Figure S4. (Continued)
